# Characterization of an APC Promoter 1B deletion in a Patient Diagnosed with Familial Adenomatous Polyposis via Whole Genome Shotgun Sequencing

**DOI:** 10.12688/f1000research.6636.1

**Published:** 2015-06-26

**Authors:** Ted Kalbfleisch, Pamela Brock, Angela Snow, Deborah Neklason, Gordon Gowans, Jon Klein

**Affiliations:** 1 Department of Biochemistry and Molecular Genetics, School of Medicine, University of Louisville, Louisville, Kentucky, 40202, USA; 2 Clinical Genetics, Weisskopf Child Evaluation Center, University of Louisville, Louisville, Kentucky, 40202, USA; 3 Huntsman Cancer Institute, Salt Lake City, Utah, 84112, USA; 4 Division of Genetic Epidemiology, Department of Internal Medicine, University of Utah, Salt Lake City, Utah, 84112, USA; 5 Department of Medicine, School of Medicine, University of Louisville, Louisville, Kentucky, 40202, USA

**Keywords:** APC, Familial Adenomatous Polyposis, Clinical Sequencing, Next Generation Sequencing

## Abstract

Recently, deletions have been identified and published as causal for Familial Adenomatous Polyposis in the 1B promoter region of the APC gene.  Those deletions were measured using multiplex ligation-dependent probe amplification.  Here, we present and characterize an ~11kb deletion identified by whole genome shotgun sequencing.  The deletion occurred in a patient diagnosed with Familial Adenomatous Polyposis, and was located on chr5, between bases 112,034,824 and 112,045,845, fully encompassing the 1B promoter region of the APC gene.   Results are presented here that include the sequence evidence supporting the presence of the deletion as well as base level characterization of the deletion site.  These results demonstrate the capacity of whole genome sequencing for the detection of large structural variants in single individuals.

## Introduction

Familial Adenomatous Polyposis (FAP) is an autosomal dominant condition characterized by the development of hundreds to thousands of polyps in the colon. This condition results in colon cancer in adult individuals in their late 20s to early 30s with nearly 100 percent penetrance. Mutations in two genes, the adenomatous polyposis coli (APC) and mutY homolog (MUTYH) loci, have been identified as causative for this disease. The majority of the mutations occur in the APC locus. The APC mutations often take the form of single nucleotide substitutions or small insertions or deletions in the coding region of the gene that produce premature stop codons, or frame shifts respectively. These result in a change of function. The exact mechanism by which these mutations affect the disease is unknown. However, deletions of APC promoter 1B are known to cause a significant change in transcription levels of the APC RNA marked by allele specific differences in transcription ^1, 2^. Several mutations have been reported in the promoter region of the APC gene ^1– 4^, identified either by sequencing, or by multiplex ligation-dependent probe amplification (MLPA). 

The patient analyzed in this work is a 50 year-old Caucasian female who has a personal and maternal family history of FAP. She developed colon polyps at 14 years of age and underwent a partial colectomy at 16 years. The patient had a complete colectomy and a Whipple procedure in her 20’s. Her mother and multiple avunculars and cousins on the maternal side are affected. One sibling has a clinical diagnosis of FAP and three siblings are unaffected. The patient’s maternal grandfather died of colon cancer later in life, but a diagnosis of FAP was not confirmed.

Previously, DNA testing in family members had failed to identify a causative mutation. Therefore, the patient and her family participated in a linkage analysis project through the Mayo Clinic in Rochester, Minnesota to identify at-risk family members. The FAP in the family showed linkage to the APC locus on chromosome 5. The patient underwent molecular testing of the APC gene (sequence analysis and Southern blot) and MUTYH gene (analysis for 2 common mutations) in 2008. No mutations were detected. A variant of unknown significance (referred to as Glu1317Gln) was found in the APC gene. However, this variant was absent in other affected family members and was present in the patient’s unaffected child. It was later classified as likely benign ^5^. The multiplex ligation-dependent probe amplification (MLPA) assays for the APC locus in use at the time did not characterize the APC promoters, and was negative for APC mutations for this patient.

In an effort to comprehensively search for potential mutations, the patient’s genomic DNA was sent to Illumina whole genome sequencing. A deletion of ~11kb encompassing the APC promoter 1B was identified, and is consistent with the deletion identified recently by Snow *et al.* ^2^ via an updated MLPA assay for APC that now includes promoter 1B and by Lin *et al.* ^4^.

In this work, we present a comprehensive characterization of this deletion using Illumina short reads, including base level resolution of the deletion site. Further, it is demonstrated that this deletion is detectable using the MLPA assay for the APC locus current at the submission of this article, and would be ambiguous if this, or any single patient were analyzed solely via whole exome sequencing.

## Methods

### Sequencing and alignment

The whole blood sample for this study was collected under a protocol approved by the University of Louisville IRB (IRB tracking number 11.0659, approval date 1/30/2012). Written informed consent for publication of clinical details was obtained from the patient/next of kin. The blood was sent to the Illumina Clinical Services Laboratory for paired end sequencing of 100 bp reads from fragments with a target length of 300bp. The reads produced were mapped via CASAVA (CASAVA-1.9.0a1_110909) to the human reference genome build 37.1 at an average depth of coverage 37.51X.

### Remapping and variant detection

The pipeline employed in our lab for read mapping and variant detection uses the Burrows-Wheeler Alignment ^6^ algorithm, and the Genome Analysis Toolkit ^7^ respectively. To be consistent with other work in our lab, reads for the regions of interest were extracted from the bam file produced by Illumina, and run through our pipeline.

Mapped reads were extracted from the binary alignment map file for remapping using Samtools ^8^ version 0.1.18 from the individual’s full binary alignment map file (provided by Illumina) corresponding to 50,000 bases upstream and downstream of the APC, and MUTYH loci defined respectively by the mapping of accession NM_001127511.1 and NM_001293192.1 to human genome build 37.1 (chr5:111,993,219-112,231,936 and chr1:45,744,915-45,856,143). Reads mapping to other chromosomes, or positions on chromosomes 1 and 5 outside of the target region would have also been extracted if their mate mapped within the target regions. Reads in these extraneous regions were not considered in variant detection. To be consistent with the remainder of our work, the FASTQ files corresponding to the first and second reads of the pair (R1 and R2) were re-derived via BEDTools ^9^ from the BAM file provided by Illumina, and remapped using the BWA algorithm for short read alignment. Duplicates were marked, indels were realigned, base quality scores recalibrated, and variants identified and simultaneously genotyped for our trace data by applying the GATK MarkDuplicates, IndelRealigner, BaseRecalibrator, and HaplotypeCaller algorithms respectively ^10, 11^.

The deletion was identified by visual inspection within the Integrative Genomics Viewer (IGV) ^12^ of the mapped next generation sequence data set as well as the variation reported in the accompanying variant call format file. This deletion is characterized by a loss of heterozygosity of variants measured relative to the reference, a cluster of 11 paired end reads (target length 500 bases) whose mates map in excess of 11kb from one another, as well as 15 reads that span the junction of the deletion that were soft trimmed by the mapping algorithm. The option “Show soft-clipped bases” within View/Preferences/Alignments was turned on and revealed soft trimming that began in several reads at positions 112,034,824 and 112,045,845 on chromosome 5. Bases from these reads were copied from within the IGV user interface for subsequent analysis in BLAT ^13^ to confirm the position of the deletion.

### PCR and Sanger Sequencing Confirmation

Primers were designed to specifically interrogate this deletion with one primer pair flanking the deletion, and one primer pair with one primer located in the deleted region. The primer located 3’ to the deleted region was common to both pairs. Full description of the primers is in provided in Table 1.

**Table 1.** Primers used to genotype samples for the presence or absence of the deletion described in this work.

	Primer Pair 1	Primer Pair 2
Left Primer	GGGCTAGTTCATTCGTTGCT	CACACCTACCATTGTGTTACCATT
Right Primer	GAGGGGGTTGCTCTTGAAA	GAGGGGGTTGCTCTTGAAA
Product Length-No Deletion	1058 Bases	11653 Bases
Product Length-With Deletion	No Product	653 Bases

### DNA extraction, PCR, and Sanger Sequencing

Whole blood was fractionated by spinning at 5,000 rpm for 10 minutes at room temperature. White cells were transferred to sterile, nuclease free microcentrifuge tubes and stored at -20°C until processing. Genomic DNA was isolated from 250uL buffy coat with Gentra Puregene Genomic DNA purification buffers (Qiagen, Valencia, CA). Separate amplification of the wild type or deletion APC fragments were performed in a 20uL reaction containing 0.4uL Phusion HF DNA Polymerase (Thermo Fisher Scientific, Pittsburg, PA), 1x Phusion Reaction Buffer, 200uM dNTP’s (Promega Corporation, Madison, WI), 200ng gDNA, and 0.5uM each primer. The cycling conditions were as follows: 98°C for 30s followed by 35 cycles of 98°C for 10s, 60°C for 30s, and 72°C for 60s, ending with a final extension of 72°C for 7min.

The amplicons were sequenced with BigDye® Terminator v3.1 (Life Technologies Corporation, Carlsbad, CA) utilizing the PCR primers and standard sequencing conditions. The sequence reactions were purified with Performa DTR Ultra 96-well filtration plates (Edge Biosystems, Gaithersburg, MD) and processed on the ABI 3130xl Genetic Analyzer (Life Technologies Corporation, Carlsbad, CA).

**Figure 1.**
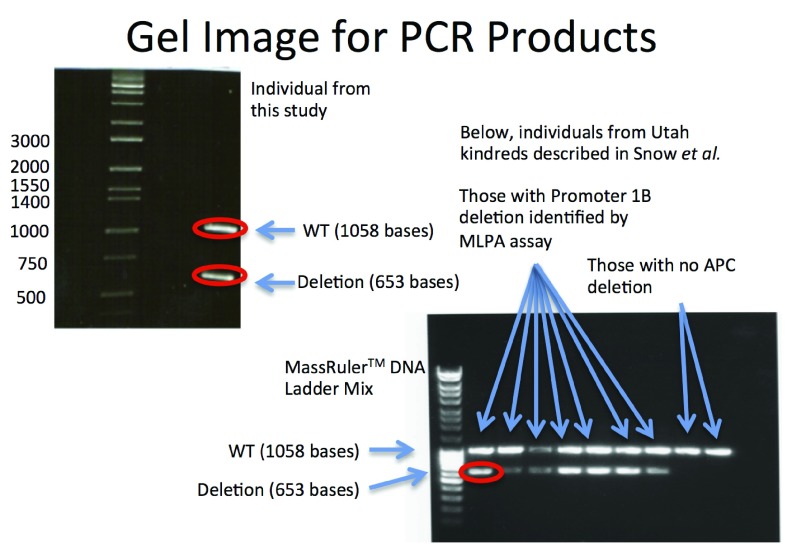
Gel images for the PCR products produced in the Louisville Index case, as well as the seven kindreds reported by Snow *et al.*, using the two primer pairs described in Table 1. The relationship between the lanes and the nine kindreds defined in Snow *et al.* are Lane 1: Ladder, Lane 2: Kindred 8, Lane 3: Kindred 43, Lane 4: Kindred 44, Lane 5: Kindred 256, Lane 6: Kindred 509. Lane 7: Kindred 685, Lane 8: Kindred 691, Lane 9: Kindred 353 (APC c.426_427delAT) And Lane 10: Kindred 6699 (APC c.532–941G>A). These images demonstrate heterozygous deletions in eight of the samples analyzed. PCR products corresponding to the bands circled in red were sequenced using Sanger technology. Those results are shown in Figure 2.

**Figure 2.**
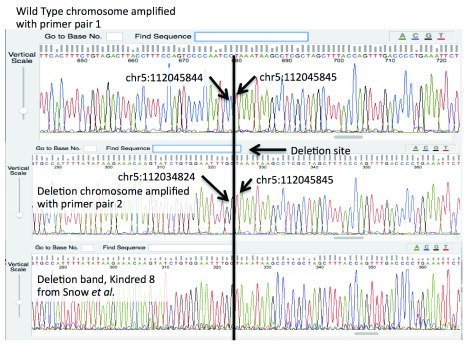
Results from Sanger sequencing for both alleles in the Louisville patient, as well as the deleted allele in a patient from the study of Snow *et al.* The top two traces indicate the nucleotide sequence of the wild type APC locus in the Promoter 1B region, and the deletion site. The bottom trace demonstrates that the deletion detected by Snow *et al.* is identical to the deletion to the individual described in this work.

The resulting gel for the PCR products is shown in Figure 1, and the sequencing results are shown in Figure 2, rendered in Geospiza’s FinchTV, ( http://www.geospiza.com/Products/finchtv.shtml).

Raw Gel electrophoresis image for Figure 1 The gel image represented in Figure 1, showing in lane 1 a control human sample that was not part of this work which was cropped from the in-text figure ^15^.

## Results

Paired end whole genome sequence data was generated at ~40X coverage for the patient, and mapped to the human reference assembly Build-37.1. Given the clinical phenotype our initial analysis of the data was limited to the APC and MUTYH loci. Variation analysis was performed in the region defined by the 5’ and 3’ most exons of the longest reported transcript for APC and MUTYH, plus and minus 50,000 bases respectively (described in detail in Methods). The resulting counts of single nucleotide variations (SNVs) and small indels are shown in Table 2– Table 4. The corresponding VCF file, along with mapped reads for these regions are available for download or visualization at http://dx.doi.org/10.13013/J6QN64N8. When viewing in IGV, navigate to the APC locus by entering APC in the text box at the top of the frame.

**Table 2.** Single nucleotide variants (SNVs) identified in our patient for the APC and MUTYH loci.

Gene	SNVs	Intronic	5’ UTR	In Coding Region			3’ UTR
				Silent	Missense	Nonsense	
APC	154	143	2	6	2	0	1
MUTYH	7	5	0	1	1	0	0

**Table 3.** Insertions and Deletions identified in our patient for the APC and MUTYH loci.

Gene	In/Dels	Intronic	5’ UTR	Frame Shifting	3’ UTR
APC	26	25	1	0	0
MUTYH	3	3	0	0	0

**Table 4.** Positions of the missense variants detected in the MUTYH and APC loci. Single Nucleotide Polymorphism database (dbSNP) accession numbers and Human Genome Variation Society (HGVS) names for the gene, including the amino acid change and position are also listed.

Gene	Chr	Coordinate	Reference Allele	Variant Allele	dbSNP	HGVS Names
MUTYH	1	45800156	C	T	rs3219484	NP_001041636.1 Val22Met
APC	5	112175240	G	C	rs1801166	NP_000029.2 Glu1317Gln
APC	5	112176756	T	A	rs459552	NP_000029.2 Val1822Asp

**Figure 3.**
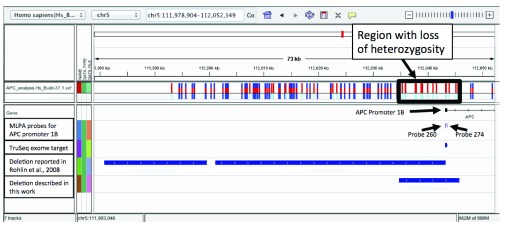
A rendering of human chromosome 5 with features between bases 111,978,904 and 112,052,349 for human genome build 37.1 in the Integrative Genomics Viewer. Records from the VCF file for the patient described here are displayed in the top track indicating a region with a loss of heterozygosity consistent with a deletion. We also render the exon identified as APC promoter 1B, the MLPA probes used commercially to analyze this locus, the region selected for pull-down in the TruSeq exome capture kit, the deletion reported by Rohlin *et al.* in 2008, and the position of the deletion described here relative to all these features.

All missense variants identified had corresponding records in dbSNP and are listed in Table 4. None are reported as deleterious. There were no non-sense SNVs or frame shifting small insertions or deletions identified. The search was then turned toward larger structural variants. Visual inspection of the VCF file for the APC locus revealed a region of approximately 10kb with 17 measured SNVs or small insertions relative to the reference. None of their respective genotypes were classified as heterozygous. This loss of heterozygosity suggested a deletion. Upon further inspection, there were other signatures characteristic of a deletion, that included a cluster of paired end reads whose mate mapped ~11kb from their respective start, and several mates that were soft trimmed because they spanned the deletion site. These soft trimmed mates were identified (described in methods), and aligned via BLAT to hsBuild-37.1, revealing the deleted region to be of length 11,020 bases, located on chr5, between bases 112,034,824 and 112,045,845, spanning the annotated APC promoter 1B. This deletion is illustrated in Figure 3, along with the positions of commercial probe sets, and other annotation relevant to this work. Given that this deletion was consistent with the deletion reported by Snow *et al.*, the primers used for verification in this work, were run on the kindreds studied in that work. It was verified that the deletion reported there was identical to the one reported here. Also, this deletion is identical to a deletion published by Lin *et al.*, ^4^ identified in kindreds from Missouri, Illinois, and Idaho not known to be related to each other.

The Illumina paired end short read data that provides evidence for the deletion relative to the reference has been isolated from the larger dataset, and is made available in its own binary alignment map file for inspection at the DOI included above.

In order to confirm the deletion, PCR primers were designed to specifically interrogate it. These primers produce a product of approximately 1kb for individuals with no deletion, and a second pair of primers was designed that flank the deletion site. This placement produces a product of 0.6kb from chromosomes with the deletion, and 11.7kb in chromosomes without. As the NGS data suggests a heterozygous deletion, the expectation was a single band with the first primer pair, and two bands, one strong from the 0.6kb amplicon, and one weak (if detectable) for the 12kb amplicon. This was confirmed in the gel represented in Figure 1. The ~1kb and .6kb bands were cut from the gel and sequenced using Sanger technology. The trace images are shown for the two different alleles in Figure 2. One read shows the deletion, and the second allele is consistent with the reference. The deletion is confirmed by the Sanger sequence data, and the primers are provided as a definitive Sanger sequencing assay for it. The second PCR image in Figure 1, and third read included in Figure 2 confirmed that our respective kindred shares the same deletion as the seven families reported by Snow *et al.* We predict that all families descend from a common founder.

Although this deletion was identified by visual inspection, the binary alignment map file for the region was analyzed by the application BreakDancer ^14^ to determine if the deletion could be identified algorithmically from whole genome sequence data. BreakDancer identifies putative deletions by identifying read pairs, clustered by genomic coordinate, that have similar inferred insert sizes which are either much larger or smaller than the standard distribution of insert sizes measured for mapped pairs. Using this algorithm, a deletion was identified on chr5 and was approximated to lie between bases 112,034,793 and 112,045,844, corroborating the finding presented here.

The methods of Snow *et al.* used multiplex ligation-dependent probe amplification (MLPA) assays. These are described in a document from MRC-Holland, available at the time of publication at ( http://www.mlpa.com/WebForms/WebFormDBData.aspx?FileOID=McLO2Mc0V%5Cc%7C). Information for those probes, including the partial sequence adjacent to the ligation site, as well as the genomic coordinate derived from a BLAT search using the partial sequence information is reproduced in Table 5, and rendered in Figure 3 relative to the deletion identified in this work. These coordinates are contained within the region deleted for this patient, and as such result in a deletion of the signals corresponding to these probes. The next probe in the set, APC 142, which is outside the deleted region, did not indicate a deletion.

**Table 5.** Probe sequence and genomic coordinate information for the MLPA probes that interrogate APC Promoter 1B.

APC Probe	Partial sequence adjacent to the ligation site	Genomic Coordinate
260	GCATTGTAGTCT-TCCCACCTCCCA	chr5:112,043, 195-112,043,218
274	TACTTCTGGCCA-CTGGGCGAGCGT	chr5:112,043, 549-112,043,572

## Discussion/Conclusion

Several years ago, a female patient of the University of Louisville Weisskopf Child Evaluation Center presented with Familial Adenomatous Polyposis (FAP). Whole genome shotgun sequencing on the Illumina platform revealed a deletion on chromosome 5 between bases 112,034,824 and 112,045,845, fully encompassing promoter 1B of the APC locus. Deletions that include this promoter have been demonstrated to affect the expression of the full length APC transcript.

In other work by Snow *et al.*, a deletion was identified via MLPA that is consistent with the deletion characterized here. An investigation via PCR of their seven kindreds with the primers used in this work establishes that the deletion is identical to the deletion reported here. Furthermore, this deletion is also reported by Lin *et al.*, in three kindred not known to be related to each other, or these families. It is likely that this mutation descends from an ancestor common to each of these reported families.

Exome capture has become a popular tool for mutation screening in clinical genetics. The deletion reported here extends several kilobases beyond the region captured by one of the more popular exome capture products ( Figure 3). This deletion would have been very difficult to identify by exome capture since the only practical measurements that could have been employed would have been read density and loss of heterozygosity in the captured region.

The whole genome sequencing approach taken here produces an information rich dataset capable of resolving large deletions in individuals. These structural variants result in a number hallmarks that are easily detected. Specifically, the loss of heterozygosity over a large region, a collection of read pairs whose mates consistently map much further apart than the majority of the read pairs, and soft trimmed reads all pinpoint the deletion site unequivocally. We have demonstrated that whole genome sequencing is both a sensitive and accurate approach for the detection and characterization of deletions of this size.

## Data availability

*F1000Research*: Dataset 1. Raw Gel electrophoresis image for Figure 1, 10.5256/f1000research.6636.d50276 ^15^
