# Supplementary figures and images for: Characterization of an APC Promoter 1B deletion in a Patient Diagnosed with Familial Adenomatous Polyposis via Whole Genome Shotgun Sequencing
Source: F1000Res. 2015 Jun 26;4:170. [Version 1] doi: 10.12688/f1000research.6636.1 (PMC4505784; doi:10.12688/f1000research.6636.1)

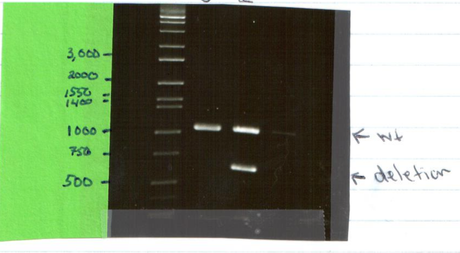

Supplement: Raw Gel electrophoresis image for Figure 1 — The gel image represented in Figure 1, showing in lane 1 a control human sample that was not part of this work which was cropped from the in-text figure 15. [file f1000research-4-7129-s0000.tgz › 2e41d062-e3a2-4f5c-ad0e-65d70646781d_Supplemental_Figure.tiff]
