# Supplementary material for: Characterization of an APC Promoter 1B deletion in a Patient Diagnosed with Familial Adenomatous Polyposis via Whole Genome Shotgun Sequencing
Source: F1000Res. 2015 Jun 26;4:170. [Version 1] doi: 10.12688/f1000research.6636.1 (PMC4505784; doi:10.12688/f1000research.6636.1)
Supplement: Peer review history

# Peer review: Referee response for version 1

**DOI:** 10.5256/f1000research.7129.r9224

**Published:** 2015-07-15

**Authors:** Anna Rohlin

**Affiliations:** 1 Department of Medical and Clinical Genetics, University of Gothenburg, Gothenburg, Sweden

Kalbfleisch *et al. *present an interesting article on identification and characterization of an 11kb *APC* promoter deletion from whole genome sequencing (WGS) data. The design of the study, the methods used and the presentation of the results are presented in a conclusive and way suitable to the investigation. They have analyzed the WGS data only for the *APC* and *MUTYH* locus and identified the deletion by visual inspection of the data. The identified deletion is confirmed and breakpoints identified with sanger sequencing. The deletion is also found to be identical to the previously identified deletions by Snow *et al.* and Lin *et al.,* and they all descent from a common founder.

**Minor points for revision and comments:**

1. When a coding DNA reference sequence is used the following recommendations can be followed according to HGVS; the coding DNA reference sequence should be complete and preferably derived from the *RefSeq database* *(format NM_033337.2)*. In general the longest transcripts are used, and for the *MUTYH* gene this is NM_001128425.1 (16 exon 549 aa). It would be more appropriate to at least name the variants found according to the NM reference sequence that has been used in table 4. For example the variant rs3219484 if the NM_001128425.1 would have been used the variant would preferable be named **NM_001128425.1:c.64A>G,** **p.Val22Met. **For the *APC* gene NM_000038.5 is usually used. In the tables and text when discussing genomic coordinates, the genome build used would be good too include for example (GRCh37/hg19).
2. When discussing MLPA, it would be appropriate to mentioned the versions of the MLPA kit, like P043 (version C1), this is a common way to present which kit has been used, a link to the document describing the assay is not necessary. Probes for the APC promoter 1B were included in the MLPA kit in 2011.
3. In the result section findings of missense and nonsense variants in coding region and small indels are mentioned, what about variants in splice acceptor or donor-sites are these included in the analyses done? It would be nice to mention this in the text and also in the tables (2 and 4) if anyone’s are found as these variants are important and often constitute disease-causing mutations. 
4. Regarding the deleteriousness of the variants found how was this interpreted? It would be nice to mentioned which databases and/or prediction tools has been used since all variants in dbSNP are not benign. I would be recommended to use several prediction tools like for example SIFT, Polyphen-2, Mutation taster, Condel and Combined Annotation-Dependent Depletion (CADD) among others for missense interpretation and also looking at conservation between species.Other important tools used for interpretation of variants found are databases were variants are reported and sometimes also classified often in a 1-5 scale (1 is benign and 5 pathogenic). The InSiGHT database ( http://insight-group.org/variants/database/) is commonly used for variants in coloncancer genes, where the *APC* and *MUTYH* gene can be found among others, clinvar and HGMD professional (Human Genome Mutation Database) are also databases too use. Information about the minor allele frequency of the variant found can also be found in ExAc (Exome Aggregation Consortium) and ESP (Exome Sequencing Project) and can be included when reporting variants.
5. This deletion was found by manual inspection of the region were the reads maps further apart than expect and by looking at the soft-clipped bases as well as identifying the region having LOH. It would be interesting to discuss the limitation with this visual method regarding sizes and type of rearrangement that can be detected. Some discussion comparing (pros and cons) regarding this method with different algorithms methods like BreakDancer (read-pair methods) and others including read-depth methods and split reads methods for example would also be valuable for an interesting discussion useful for readers trying to find methods to analyze for these types of mutations.

I have read this submission. I believe that I have an appropriate level of expertise to confirm that it is of an acceptable scientific standard.

# Peer review: Referee response for version 1

**DOI:** 10.5256/f1000research.7129.r9222

**Published:** 2015-07-08

**Authors:** Nicholas Davidson, Yiing Lin

**Affiliations:** 1 Division of Gastroenterology, Washington University in St. Louis, St. Louis, MO, USA; 2 Department of Surgery, Washington University in St. Louis, St. Louis, MO, USA

Mutations in the *APC* gene have been established as a cause of classical FAP. However, in a small subset (~20%) of affected families, mutations in the coding regions of *APC* or other polyposis-associated genes such as *MUTYH* cannot be identified. In this study, the authors perform whole-genome sequencing on one such subject and identify a heterozygous 11kb deletion in the exon 1B / promoter region of APC. The three non-synonymous mutations identified in *APC* and *MUTYH* were not felt to be causal mutations. The authors confirmed the presence of the heterozygous deletion through differential PCR and Sanger sequencing.

The authors then went on to investigate nine APC mutation-negative kindreds which were previously found to have exon 1B / promoter deletions using multiplex ligation-dependent probe amplification assays. In all nine kindreds, the same coordinates of the 11kb deletion were inferred.

The study design, presentation of results and conclusions are straightforward. The authors find an *APC* promoter deletion through whole-genome sequencing and establish the deletion coordinates through Sanger sequencing. Ideally, further investigation of a number of affecteds and unaffected members in the kindred of this study would provide convincing evidence that this 11kb deletion is associated with the affected state. However, considered in conjunction with the results from Lin *et al.* and Snow *et al.*, this study shows that whole-genome sequencing is a suitable method for the detection of non-coding mutations in *APC*-mutation negative FAP individuals. This builds upon mounting evidence that associates *APC* promoter deletions with FAP. Interestingly, these studies all show that affected members of eleven FAP kindreds in the United States share the promoter deletion with identical coordinates. It might be worth noting in the discussion that the original Snow *et al *paper using MPLA identified a deletion that was thought to be much larger (>33kb) but that approach did not map the exact coordinates.

Two minor points: (1) In the Results section (p 6 left column second paragraph), it is stated: “these primers produce a product of approximately 1kb for individuals with no deletion.” More accurately, the 1kb product is produced from chromosomes without the deletion (as the authors later state); the 1kb product is also produced in all individuals with the deletion (Fig. 1).

(2) In the discussion, the authors discuss potential difficulties in using targeted capture techniques to discover or assess larger deletions such as the one described. However, we successfully used such a targeted capture assay to discover the promoter deletion described in our study. The analysis methods for detecting individual reads and read pairs that straddle the deletion are applicable and to targeted sequencing strategies.

We have read this submission. We believe that we have an appropriate level of expertise to confirm that it is of an acceptable scientific standard.
